# Circulating exosome long non-coding RNAs are associated with atrial structural remodeling by increasing systemic inflammation in atrial fibrillation patients

**DOI:** 10.2478/jtim-2023-0129

**Published:** 2024-03-21

**Authors:** Yue Yuan, Xuejie Han, Xinbo Zhao, Haiyu Zhang, Asiia Vinograd, Xin Bi, Xiaoxu Duan, Yukai Cao, Qiang Gao, Jia Song, Li Sheng, Yue Li

**Affiliations:** Department of Cardiology, the First Affiliated Hospital, Harbin Medical University, Harbin 150001, Heilongjiang Province, China; Bashkir State Medical University, UFA, Republic Bashkortostan, Russia; Department of Medicine, Division of Atherosclerosis and Vascular Medicine, Baylor College of Medicine, Houston 77054, USA; NHC Key Laboratory of Cell Transplantation, Harbin Medical University, Harbin 150001, Heilongjiang Province, China; Key Laboratory of Hepatosplenic Surgery, Harbin Medical University, Ministry of Education, Harbin 150001, Heilongjiang Province, China; Heilongjiang Key Laboratory for Metabolic Disorder & Cancer Related Cardiovascular Diseases, Harbin 150081, Heilongjiang Province, China; Key Laboratory of Cardiac Diseases and Heart Failure, Harbin Medical University, Harbin 150001, Heilongjiang Province, China

**Keywords:** exosomes, lncRNAs, atrial fibrillation, inflammation, structural remodeling

## Abstract

**Background:**

Atrial fibrillation (AF) is the most common cardiac arrhythmia with severe clinical sequelae, but its genetic characteristic implicated in pathogenesis has not been completely clarified. Accumulating evidence has indicated that circulating exosomes and their carried cargoes, such as long non-coding RNAs (lncRNAs), involve in the progress of multiple cardiovascular diseases. However, their potential role as clinical biomarkers in AF diagnosis and prognosis remains unknown.

**Methods:**

Herein, we conducted the sequence and bioinformatic analysis of circulating exosomes harvested from AF and sinus rhythm patients.

**Results:**

A total of 53 differentially expressed lncRNAs were identified, and a total of 6 significantly changed lncRNAs (fold change > 2.0), including NR0046235, NR003045, NONHSAT167247.1, NONHSAT202361.1, NONHSAT205820.1 and NONHSAT200958.1, were verified by qRT-PCR in 215 participants. Moreover, these circulating exosome lncRNA levels were different between paroxysmal and persistent AF patients, which were dramatically associated with abnormal hemodynamics and atrial diameter. Furthermore, we observed that the area under ROC curve (AUC) of six lncRNAs combination for diagnosis of persistent AF was 80.34%. Gene ontology (GO) and Kyoto encyclopedia of genes and genomes (KEGG) enrichment pathway analysis indicated these exosome lncRNAs mainly concerning response to chemokine-chemokine receptor interaction, which induced activated inflammation and structural remodeling. In addition, increased plasma levels of CXCR3 ligands, including CXCL4, CXCL9, CXCL10 and CXCL11, were accumulated in AF patient tissues.

**Conclusion:**

Our study provides the transcriptome profile revealing pattern of circulating exosome lncRNAs in atrial structural remodeling, which bring valuable insights into improving prognosis and therapeutic targets for AF.

## Introduction

Atrial fibrillation (AF) is the most prevalent cardiac arrhythmia in adults, with a prevalence of 2%-4% in the general population.^[1]^ As one of the aged related diseases, it can rise up to 8%-12% in the elderly.^[2,3]^ AF contributes to a 2-fold increase in cardiovascular morbidity and mortality, thus leaving a tremendous burden on patients and societal healthcare.^[4,5]^ AF is progressive in nature, and most patients progress from paroxysmal to persistent AF, even permanent AF.^[6]^ The pathophysiology involved in AF has been investigated for almost a century, but the exact underlying mechanisms remain unclear. Atrial remodeling is the crucial substrate in AF pathogenesis, which increases the risks of heart failure and thromboembolic events.^[7]^ Therefore, further interpretation is urgently needed to ameliorate the prevention strategy of AF.

A growing body of evidence has suggested genetic cause for encoding ion channels, inflammation and fibrosis during AF.^[8]^ Recently, next-generation sequencing technologies enlighten that non-coding RNAs (ncRNAs) emerge as novel epicenter for the development of cardiovascular diseases (CVDs).^[9]^ LncRNAs are characterized as non-protein coding RNAs longer than 200 base pairs in length, which play crucial roles in the development of CVDs. Several studies have revealed differentially expressed lncRNAs in atrial tissues and peripheral circulating monocytes of AF patients.^[10,11]^ However, it is still lack of available clinical biomarker for earlier diagnosis, prognosis and precise treatment of AF.

In the past decade, seminal researches confirm that extracellular vesicle (EV) are representing as a new mean of cell-to-cell crosstalk, which participate in multiple physiological processes, including cell death, angiogenesis and immune response.^[12]^ Exosomes, a classical subtype EVs, defined as 30–150 nm membrane-bound vesicles, are originated from endosomes and considered to have the potential for the diagnosis of diseases.^[13]^ Indeed, circulating exosomes and conveyed cargoes have gained attention as more stable clinical biomarkers for diagnosis and prognosis of various CVDs.^[14]^ Recent data underlines that circulating exosome lncRNAs provide several advantages to reflect disease state and predict progression.^[15]^ However, the circulating exosome lncRNA profile and their role in AF has not been clarified.

In the current study, we attempted to reveal circulating exosome lncRNAs and mRNAs profile during the pathogenesis of AF via transcriptome sequencing. Several differential exosome-loaded lncRNA expressions were verified by qRT-PCR in expanded patient-based samples. We subsequently established lncRNA-mRNA network to seek crucial candidate genes and explore their roles in the progress of atrial remodeling and the potential mechanisms. It is believed that circulating exosome lncRNA can serve as available molecule for clinical applications and uncover advancing knowledge on the mechanism of AF.

## Materials and methods

### Patients in the study

All individuals in our study were recruited from the Department of Cardiology, the First Affiliated Hospital in Harbin Medical University, as a single institution from September 2018 to June 2021. The study has been approved and registered by the Ethics Committee in the First Affiliated Hospital of Harbin Medical University (No. 201868). The informed consent was obtained from all participants. The details of included and excluded criteria for all enrolled individuals were provided in the Supplemental section.

### Exosome lncRNA Sequence and bioinformatic analysis

The plasma exosome of human blood samples was lysed by Trizol and the RNA content was extracted. Then, lncRNA and mRNA sequencing was performed at Guangzhou RiboBio Co., Ltd. with the Illumina HiSeq 3000.^[16]^ Differential expression analysis of the RNA-seq was examined according to a fold-change (FC) ≥ 2.0 and *P* value < 0.05. The bioinformatic analysis of differential exosome lncRNAs was performed through Gene Ontology (GO) categories and Kyoto Encyclopedia of Genes and Genomes (KEGG) database. Then the downstream target genes regulated by above differential lncRNAs were predicted by starBase v2.0. The bioinformatic analysis of predicted downstream target genes was performed through GO categories and KEGG database including cis and trans function.

### Animal study

All animal experiment protocol was approved by the Ethics Committee in the First Affiliated Hospital of Harbin Medical University. SD rats were used to establish the exosome transfer model at 8-weeks-old age. Exosomes (1–5×10^10^ diluted with 200 μL PBS) were transferred to rat via tail vein injection every other day for 7 times. Exosomes injected in each rat were purified from sinus rhythm (SR) or AF individual plasma. Inducible AF was performed with essentially the same protocol as described previously in detail.^[4]^ Briefly, rats underwent open-chest electrophysiological programmed stimulation under 1% sodium pentobarbital (30 mg/kg) anesthesia. The 1.9-F octapolar catheter (Transonic Systems Inc, New York, USA) was placed on the right atria for programmed stimulation. To assess AF inducibility, 50-Hz burst pacing was applied for 3-second with 12 bursts separated by a 2-second interval. AF was defined as ≥ 1 second of irregular atrial electrograms ( > 800 beats/min) with irregular ventricular response. AF duration was defined as the mean duration of all AF episodes within 60 seconds.

### Statistical analysis

Normality test was performed to determine whether the quantitative data were normally distributed. Nonparametric test and/or spearman correlation were used when the data were not normally distributed. Continuous variables were expressed as the mean ± SD. Unpaired Student’s t-test was used to determine the significant difference between two groups. Categorical data were presented with count and percentile. Chi-square test or Fisher’s exact test was performed to analyze the categorical data. GraphPad8.0 was performed to above analysis. Area under the receiver operating characteristic (AUC) was calculated by receiver operating characteristic (ROC) curve to evaluate the predictive performance of biomarkers for clinical AF type via SPSS19.0. The other analyses were performed using R 4.0.3 software, and a two-tailed *P* < 0.05 was considered statistically significant.

Other detail information was clarified in Supplemental materials.

## Results

### Profile of differential circulating exosome lncRNAs and mRNAs in AF patients

**Figure 1:**
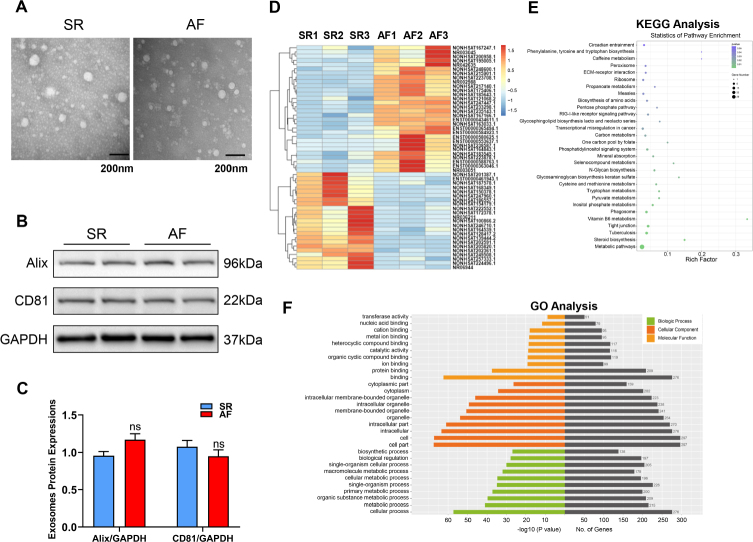
Profile of differential circulating exosome lncRNAs and mRNAs in AF patients. (A) Transmission electron microscope to observe the morphology of plasma exosomes in SR (*n* = 3) and AF patients (*n* = 3). Scale bar: 200 nm. (B) Western blot of Alix and CD81 protein levels in circulating exosomes. (C) Quantification of each protein (*n* = 4), GAPDH as an internal control. n represents the number of patients in every group. (D) Heatmap of differential circulating exosome lncRNAs between SR (*n* = 3) and AF (*n* = 3) patients. (E) KEGG pathway analysis of differential circulating exosomal lncRNAs. (F) GO functional analysis of differential circulating exosomal lncRNAs. n represents the number of patients in every group. SR: sinus rhythm, AF: atrial fibrillation.

In our study, three SR individuals and three AF patients were enrolled to lncRNA sequence, and their medical histories were recorded. The clinical details were shown in Supplemental Table 1, and there were no significant differences between two groups. The plasma samples were collected and circulating exosomes were extracted by ultracentrifugation. The form of exosomes was identified by EM, which suggested diameter of the exosome samples that did not exceed 150 nm with double membrane (Figure 1A). Besides, the specific protein markers of exosomes such as CD81 and Alix were confirmed by western blot (Figure 1B and C) in 4 SR and 4 AF patients’ plasma samples. To reveal the profile of circulating exosome lncRNAs and mRNAs, we conducted the transcriptome sequencing and 53 differentially expressed plasma exosome lncRNAs were identified, including 30 upregulated and 23 downregulated lncRNAs (Figure 1D). GO and KEGG pathway analysis showed that those differential exosome lncRNAs were mainly enriched in the pathophysiological process in molecule binding (such as RNA, protein and ion), cellular metabolism, inflammation and coagulation (Figure 1E and F). Moreover, the different exosome mRNAs between two groups were displayed in Supplemental Figure 1A. GO and KEGG pathway analysis indicated that differential exosomal mRNAs were involved in regulating protein kinase activity and binding, such as MAPK, Notch and mTOR signaling pathways (Supplemental Figure 1B and C). All above biological progresses have suggested to promote the occurrence and maintenance of AF.

### Validation of differential exosome lncRNAs in 215 individuals

**Figure 2:**
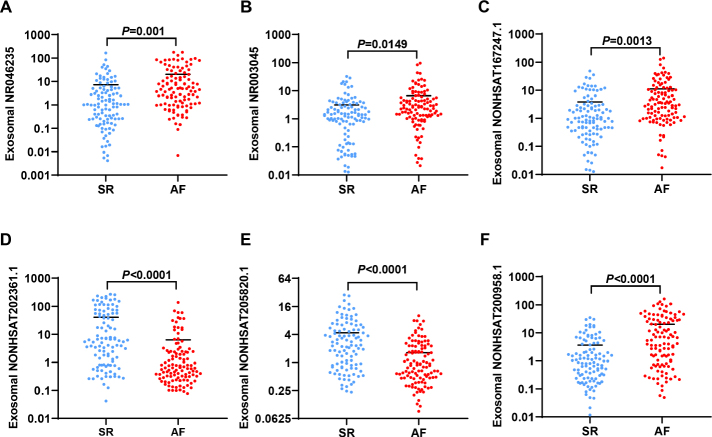
Validation of 6 differential exosomal lncRNAs in 215 individuals. (A) Plasma exosomal levels of NR046235 in SR (*n* = 105) and AF patients (*n* = 110) tested by qRT-PCR. (B) Plasma exosomal levels of NR003045 in SR (*n* = 105) and AF patients (*n* = 110) tested by qRT-PCR. (C) Plasma exosomal levels of NONHSAT167247.1 in SR (*n* = 105) and AF patients (*n* = 110) tested by qRT-PCR. (D) Plasma exosomal levels of NONHSAT202361.1 in SR (*n* = 105) and AF patients (*n* = 110) tested by qRT-PCR. (E) Plasma exosomal levels of NONHSAT205820.1 in SR (*n* = 105) and AF patients (*n* = 110) tested by qRT-PCR. (F) Plasma exosomal levels of NONHSAT200958.1 in SR (*n* = 105) and AF patients (n = 110) tested by qRT-PCR. n represents the number of patients in every group. SR: sinus rhythm, AF: atrial fibrillation.

Based on the results of exosome lncRNAs profile, 6 differential lncRNAs in Top 10 (*P* < 0.05 and Log2 fold change > 2.0) were selected as candidate indicators, including four upregulated lncRNAs (NR046235, NR003045, NONHSAT167247.1 and NONHSAT200958.1) and two downregulated lncRNAs (NONHSAT202361.1 and NONHSAT205820.1). To verify the accuracy of these 6 exosome lncRNAs to be sensitive for AF patients, a total of 215 individuals were enrolled, which contained 105 SR individuals and 110 AF patients. The baseline characteristics and comparison between SR and AF groups were shown in Table 1. There was no significant difference in gender, BMI, medical history and LV ejection fraction (LVEF). The average of age, smoking, left atria (LA) and right atria (RA) diameter were higher in AF group. The plasma exosomes of all individuals were extracted and these 6 lncRNAs expressions were detected. The expressions of six lncRNAs were performed normally distribute. The qRT-PCR results showed that the levels of NR046235, NR003045, NONHSAT167247.1 and NONHSAT200958.1 were highly expressed (Figure 2A-C, Figure 2F), accompanied with decreased NONHSAT202361.1 and NONHSAT205820.1 (Figure 2D and E) levels in AF patients. These results were consistent with the RNA sequencing data of exosome lncRNAs.

**Table 1:** Clinical Characteristics of 215 individuals

Characteristics	SR group (*n* = 105)	AF group (*n* = 110)	*P* value
Age	63.36 ± 9.23	67.08 ± 10.65	0.0068**
Gender, *n* (%)			0.4834
Male	66 (62.9)	64 (58.2)	
Female	39 (37.1)	46 (41.8)	
BMI (kg/m^2^)	24.46 ± 3.22	25.23 ± 3.18	0.0818
Smoking, *n* (%)	57 (54.3)	35 (31.8)	0.0090**
Hypertension, *n* (%)	56 (53.3)	56 (50.9)	0.7221
Diabetes, *n* (%)	27 (25.7)	24 (21.8)	0.5020
Coronary heart disease, *n* (%)	59 (56.2)	50 (45.5)	0.1155
Heart Failure, *n* (%)	21 (20.0)	33 (30.0)	0.0910
Chronic kidney disease, *n* (%)	11 (10.5)	20 (18.2)	0.1079
Aspirin	64 (61.0)	21 (19.1)	<0.001***
Statin	89 (84.8)	50 (45.5)	<0.001***
β-receptor blocker	46 (43.8)	53 (48.2)	0.5203
ACEI/ARBs	37 (35.2)	53 (48.2)	0.0545
CCB	16 (15.2)	10 (9.10)	0.1670
NOAC	2 (1.9)	81 (73.6)	<0.001***
Glucose (mmol/L)	6.39 ± 2.40	5.62 ± 1.53	0.0048**
TG (mmol/L)	1.86 ± 1.07	1.55 ± 0.85	0.0170*
CHOL (mmol/L)	4.60 ± 1.33	4.18 ± 1.06	0.0109*
Cr (mmol/L)	74.76 ± 52.46	80.91 ± 41.11	0.3383
UA (mmol/L)	323.7 ± 103.7	362.2 ± 107.9	0.0083**
NT-proBNP (pg/L)	1010.0 ± 173.9	1384.0 ± 185.4	0.1438
LAD (mm)	36.53 ± 4.26	42.20 ± 6.17	<0.0001***
RAD (mm)	43.59 ± 3.61	50.08 ± 7.37	<0.0001***
LVEF (%)	58.32 ± 10.63	58.01 ± 11.47	0.8351

BMI, body mass index; ACEI, angiotensin enzyme inhibitor; ARBs, angiotensin receptor blockers; CCB, calcium channel blocker; NOAC, novel oral anticoagulant. TG, triglyceride; CHOL, cholesterol; Cr, creatinine; UA, uric acid; NT-proBNP, N-terminal pro-B-type natriuretic peptide; LAD, left atrial diameter; RAD, right atrial diameter; LVEF, left ventricle ejection fraction. (*P < 0.05, **P < 0.01, ***P < 0.001).

### Circulating exosome lncRNAs are available biomarkers for the diagnosis of persistent AF

**Figure 3:**
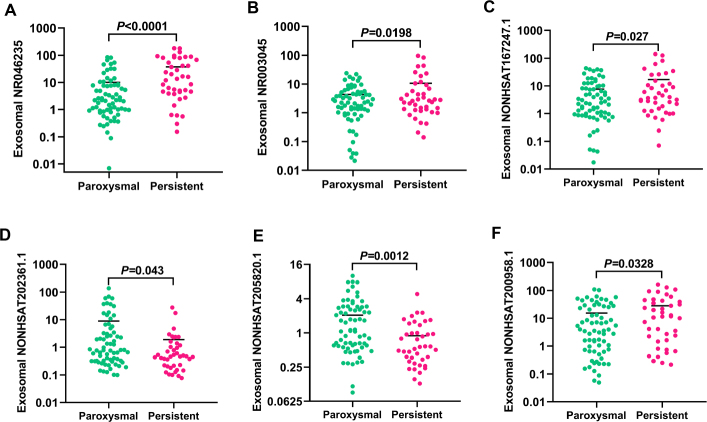
Circulating exosome lncRNAs are available as biomarkers for diagnosis of persistent AF. (A) Plasma exosomal levels of NR046235 in PAF (*n* = 69) and PeAF patients (*n* = 41) tested by qRT-PCR. (B) Plasma exosomal levels of NR003045 in PAF (*n* = 69) and PeAF patients (*n* = 41) tested by qRT-PCR. (C) Plasma exosomal levels of NONHSAT167247.1 in PAF (*n* = 69) and PeAF patients (*n* = 41) tested by qRT-PCR. (D) Plasma exosomal levels of NONHSAT202361.1 in PAF (*n* = 69) and PeAF patients (*n* = 41) tested by qRT-PCR. (E) Plasma exosomal levels of NONHSAT205820.1 in PAF (*n* = 69) and PeAF patients (*n* = 41) tested by qRT-PCR. (F) Plasma exosomal levels of NONHSAT200958.1 in PAF (*n* = 69) and PeAF patients (*n* = 41) tested by qRT-PCR. *n* represents the number of patients in every group. PAF: paroxysmal AF, PeAF: persistent AF.

**Figure 4:**
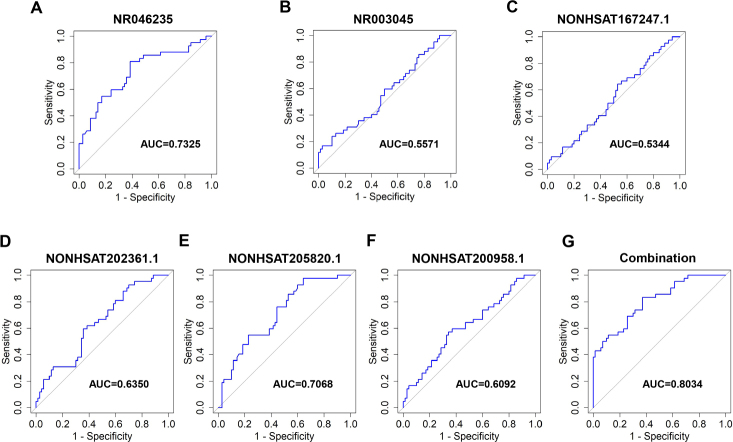
ROC curve and AUC of 6 lncRNAs for diagnosis of PeAF. (A) The ROC curve of NR046235 for diagnosis of PeAF and AUC was 0.7325. (B) The ROC curve of NR003045 for diagnosis of PeAF and AUC was 0.5571. (C) The ROC curve of NONHSAT167247.1 for diagnosis of PeAF and AUC was 0.5344. (D) The ROC curve of NONHSAT202361.1 for diagnosis of PeAF and AUC was 0.6450. (E) The ROC curve of NONHSAT205820.1 for diagnosis of PeAF and AUC was 0.7068. (F) The ROC curve of NONHSAT200958.1 for diagnosis of PeAF and AUC was 0.6092. (G) The ROC curve and AUC of combination with these 6 lncRNAs was 0.8034.

The different types of AF have great effects on atrial structure and clinical outcomes of patients. Conversely, electrical and structural remodeling promote shortening atrial effective refractory period and AF duration, which leads to so-called “AF begets AF”. Thus, early identifying clinical biomarkers of persistent AF that indicate the risk of structural remodeling will guide therapeutic strategy and reduce related complications. To this end, medical record of 110 AF patients were retrospective analyzed, and patients were divided into paroxysmal AF group (PAF, *n* = 69) and persistence AF group (PeAF, *n* = 41) based on the duration and recovering of AF. The baseline characteristics and comorbidities were summarized in Table 2. There was no statistical difference in age, gender, BMI, smoking and medical history, but PeAF patients had higher incidence of heart failure, chronic kidney disease and atrial dimension, which were consistent with previous findings. Furthermore, we explored the correlation between these 6 exosome lncRNAs and different AF clinical types. Our data showed that expressions of NR046235, NR003045, NONHSAT167247.1 and NONHSAT200958.1 were higher in PeAF group (Figure 3A-C, Figure 3F), and NONHSAT202361.1 and NONHSAT205820.1 (Figure 3D and E) performed lower levels in PeAF patients. Moreover, ROC curve and AUC were conducted to evaluate the diagnosis of 6 lncRNAs for PeAF. The AUC of NR046235, NR003045, NONHSAT167247.1, NONHSAT202361.1, NONHSAT205820.1 and NONHSAT200958.1 were respectively 0.7325, 0.5571, 0.5344, 0.6350, 0.7068 and 0.6092 (Figure 4A-F). The AUC of combination with these 6 lncRNAs was 0.8034 (Figure 4G). All the data suggests that these circulating exosome lncRNAs could be identified as potential biomarker to distinguish PeAF.

**Table 2:** Clinical characteristics of 110 AF patients

Characteristics	Paroxysmal AF (*n* = 69)	Persistent AF (*n* = 41)	*P* value
Age	66.54 ± 10.44	68 ± 11.07	0.4884
Gender, *n* (%)			0.6470
Male	39 (56.5)	25 (61.0)	
Female	30 (43.5)	16 (49.0)	
BMI (kg/m^2^)	25.18 ± 3.36	25.32 ± 2.90	0.8243
Smoking, *n* (%)	25 (36.2)	10 (24.4)	0.1973
Hypertension, *n* (%)	43 (62.3)	13 (31.7)	0.0019**
Diabetes, *n* (%)	17 (24.6)	7 (17.1)	0.3530
Coronary Heart Disease, *n* (%)	31 (44.9)	19 (46.3)	0.8855
Heart Failure, *n* (%)	3 (4.4)	30 (73.2)	<0.0001***
Chronic Kidney Disease, *n* (%)	8 (11.6)	13 (31.7)	0.0095**
Stroke, *n* (%)	13 (18.8)	11 (26.8)	0.3266
CHA_2_DS_2_VASc			
0-1	20 (29.0)	5 (12.2)	0.0422*
2	15 (21.7)	10 (24.4)	0.7483
3	23 (33.3)	13 (31.7)	0.8605
>3	11 (15.89)	13 (31.7)	0.0529
Glucose (mmol/L)	5.68 ± 1.78	5.51 ± 0.98	0.5703
TG (mmol/L)	1.62 ± 0.87	1.42 ± 0.82	0.2439
CHOL (mmol/L)	4.31 ± 1.08	3.96 ± 1.00	0.0944
Cr (mmol/L)	71.37 ± 21.47	96.96 ± 58.32	0.0013**
UA (mmol/L)	350.5 ± 93.86	381.9 ± 127.0	0.1405
NT-proBNP (pg/L)	997.3 ± 199.7	2034.0 ± 346.7	0.0063**
LAD (mm)	40.14 ± 5.30	45.68 ± 6.05	<0.0001***
RAD (mm)	47.87 ± 5.76	54.07 ± 8.53	<0.0001***
LVEF (%)	62.13 ± 7.66	51.07 ± 13.42	<0.0001***
NR046235	10.03 (18.64)	37.65 (48.03)	<0.0001***
NR003045	4.33 (5.22)	10.55 (20.79)	0.0198*
NONHSAT167247.1	7.60 (10.84)	16.98 (31.75)	0.0267*
NONHSAT202361.1	8.93 (21.63)	1.89 (4.94)	0.0430*
NONHSAT205820.1	2.05 (2.13)	0.89 (0.87)	0.0012**
NONHSAT200958.1	15.30 (23.34)	28.13 (38.91)	0.0328*

BMI, body mass index; ACEI, angiotensin enzyme inhibitor; ARBs, angiotensin receptor blockers; CCB, calcium channel blocker; NOAC, novel oral anticoagulant. TG, triglyceride; CHOL, cholesterol; Cr, creatinine; UA, uric acid; NT-proBNP, N-terminal pro-B-type natriuretic peptide; LAD, left atrial diameter; RAD, right atrial diameter; LVEF, left ventricle ejection fraction. (*P < 0.05, **P < 0.01, ***P < 0.001)

### Correlation between circulating exosome lncRNAs and atrial structure in AF patients

**Figure 5:**
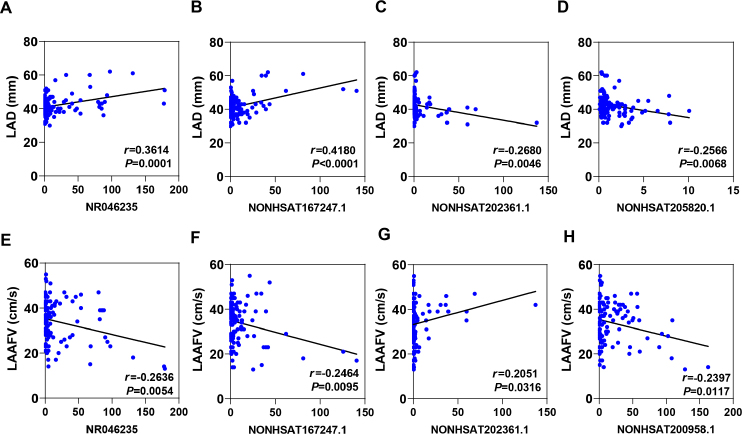
Correlation between 6 circulating exosomal lncRNAs and atrial structure and function in AF patients. (A) Pearson analysis of circulating exosomal NR046235 correlated with LAD in AF patients, *r* = 0.3614 and *P* = 0.0001. (B) Pearson analysis of circulating exosomal NONHSAT167247.1 correlated with LAD in AF patients, *r* = 0.4180 and *P* < 0.0001. (C) Pearson analysis of circulating exosomal NONHSAT202361.1 correlated with LAD in AF patients, *r* = -0.2680 and *P* = 0.0046. (D) Pearson analysis of circulating exosomal NONHSAT205820.1 correlated with LAD in AF patients, *r* = -0.2566 and *P* = 0.0068. (E) Pearson analysis of circulating exosomal NR046235 correlated with LAAFV in AF patients, *r* = -0.2636 and *P* = 0.0054. (F) Pearson analysis of circulating exosomal NONHSAT167247.1 correlated with LAAFV in AF patients, *r* = -0.2462 and *P* = 0.0095. (G) Pearson analysis of circulating exosomal NONHSAT202361.1 correlated with LAAFV in AF patients, *r* = 0.2051 and *P* = 0.0316. (H) Pearson analysis of circulating exosomal NONHSAT200958.1 correlated with LAAFV in AF patients, *r* = -0.2397 and *P* = 0.0117. LAD: left atrial diameter, LAAFV: left atrial appendage flow velocity.

A large amount of evidence reveals that enlarged LA with slow LAAFV could be a well-established predictor of adverse outcomes, such as thromboembolism.^[17]^ According to the data of echocardiography and cardiac MRI, there was a significant increase in left atria diameter (LAD) with lower LAAFV in PeAF individuals, which indicating remarkable atrial structural remodeling (Supplemental Figure 2). To elucidate whether differential exosome lncRNAs could be efficient biomarker for atrial remodeling, Pearson analysis was constructed. Surprisingly, the results showed that circulating exosomal NR046235, NONHSAT167247.1, NONHSAT202361.1 and NONHSAT205820.1 were correlated with LAD in AF group, but NR003045 and NONHSAT200958.1 showed no difference (Figure 5A-D, Supplemental Figure 3A and B). Correspondingly, we observed that plasma exosomal NR046235, NONHSAT167247.1, NONHSAT202361.1 and NONHSAT200958.1 levels also had a close association with LAAFV in AF patients, but NR003045 and NONHSAT205820.1 exhibited ineffective role (Figure 5E-H, Supplemental Figure 3C and D). Overall, these data suggest that circulating exosome lncRNA may be potential predictors of atrial structural remodeling and clinical complications in AF.

### Differential exosome lncRNA regulate CXCR3 signaling pathway resulting in inflammation and atrial structural remodeling

**Figure 6:**
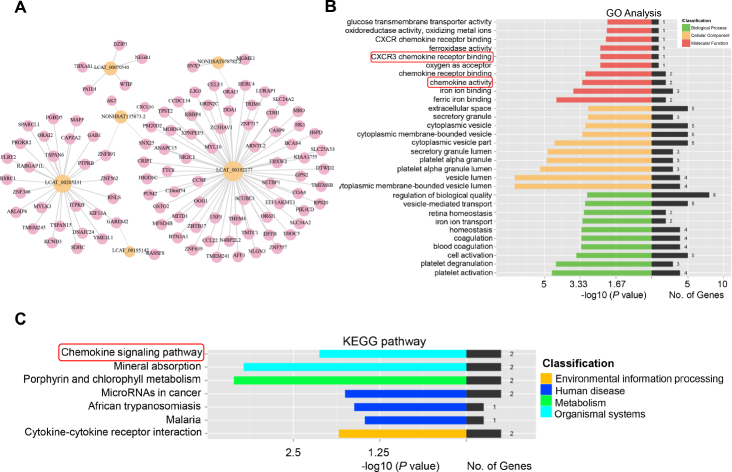
KEGG and GO analysis of target genes regulated by 6 differential exosomal lncRNAs. (A) The network of 100 target mRNAs regulated by 6 circulating exosomal lncRNAs including both cis and trans biological function. (B) GO functional analysis of 100 target mRNAs. (C) KEGG pathway analysis of 100 target genes.

**Figure 7:**
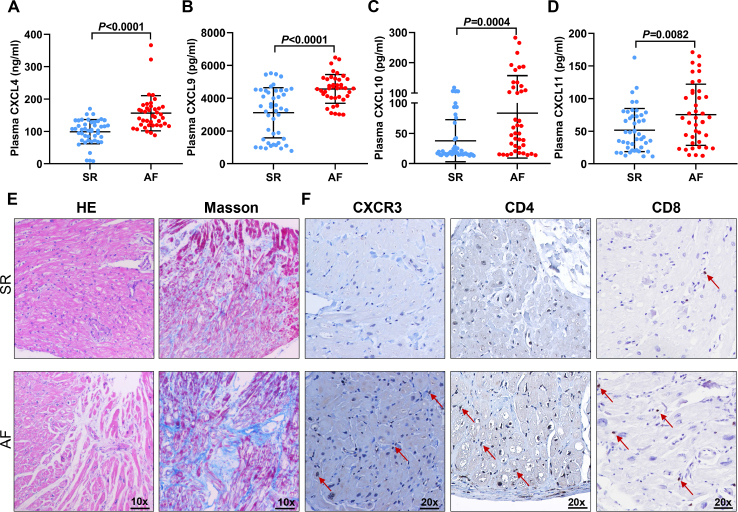
CXCR3 signaling pathway resulted in activated inflammation and atrial fibrosis. (A) Plasma levels of CXCL4 (pg/L) in SR ( *n* = 45) and AF patients ( *n* = 40). (B) Plasma levels of CXCL9 (ng/L) in SR (*n* = 45) and AF patients (*n* = 40). (C) Plasma levels of CXCL10 (ng/L) in SR (*n* = 45) and AF patients (*n* = 40). (D) Plasma levels of CXCL11 (ng/L) in SR (*n* = 45) and AF patients (*n* = 40). (E) HE and Masson staining of atrial tissues in SR (*n* = 7) and AF patients (*n* = 8). Scale bar: 100 pm. (F) IHC staining of CXCR3, CD4 and CD8 in atrial tissues in SR ( *n* = 7) and AF patients ( *n* = 8). Scale bar: 50 pm. Arrow represents positive cells. *n* represents the number of patients in every group.

We further conducted the bioinformatics analysis about these 6 lncRNAs to interpret the pathophysiological role of candidate exosome lncRNAs in the progress of AF. The network of 100 target mRNAs, including both cis and trans regulation was established (Figure 6A). Meanwhile, GO and KEGG analysis of potential genes were performed to speculate the relevant biological functions. The GO terms annotated that these lncRNAs participated in the release of chemokine, chemokine receptor binding and C-X-C motif chemokine receptor (CXCR) binding (Figure 6B and C). Among these, CXCR3 chemokine signaling has been recognized as a key pathway in regulating T cell dependent inflammation. However, its expression and ligands in atrial tissue and their role in the development of AF has not been reported. To fully verify the key role of CXCR3 in AF progress, ELISA assay was used to measure the human plasma levels of CXCL4, CXCL9, CXCL10 and CXCL11, which are the main ligands of CXCR3. Our results showed that the levels of these 4 ligands were significantly higher in AF than SR patients (Figure 7A-D). Furthermore, human atrial tissues of SR (*n* = 7) and AF (*n* = 8) individuals were collected to determine the expression of CXCR3 and its related immune cells infiltration in atrial tissues, which eventually lead to structural remodeling. As shown in HE and Masson staining, AF group has more inflammation and collagen accumulation in the atrium (Figure 7E). Then, the immune markers, including CXCR3, CD3, CD4 and CD8 were tested. Immunohistochemistry results showed significantly upregulated expressions of CXCR3 in atrial tissues of AF group. Besides, aggregated CD4^+^ and CD8^+^ T cells were observed in atrial tissues of AF patients, which was consist with structural change (Figure 7F). Taken together, these data demonstrates that CXCR3 pathway induces T cell dependent atrial inflammation and plays a key role in promoting structural remodeling during AF.

### Transfer of circulating exosome from AF patients enhanced AF susceptibility in vivo

To validate the role of circulating exosomes in AF development and test the expressions of target exosome lncRNAs, we established rat model via transferring plasma exosomes from SR and AF individuals (Supplemental Figure 4A). Then PKH26 staining was performed to observe the uptake of exosomes into rat atrium. The result showed the PKH26-labeling exosomes entered atrial cardiomyocytes in two groups (Supplemental Figure 4B). qRT-PCR suggested that the expressions of 6 lncRNAs significantly changed in the atrial tissues of Exo-AF rat compared with Exo-SR group, which was in accordance with the human data (Supplemental Figure 4C). These data demonstrated that 6 circulating exosome lncRNAs from human plasma could be absorbed by rat atrial cardiomyocytes.

Furthermore, we performed intracardiac electrophysiology to test the inducible AF incidence in two groups of animals. The results showed that the circulating exosomes from AF patients enhanced the AF susceptibility of rats compared with Exo-SR group (Supplemental Figure 5A and 5B). AF duration was performed by the nonparametric test because of its non-normal distribution. We found that the AF duration in Exo-AF group markedly prolonged compared to Exo-SR rats (Supplemental Figure 5C). Besides, we tested the protein levels of NLRP3, Caspase1, CXCR3, Collagen1 and a-SMA in two groups of rats by western blot. The results indicated that atrial inflammasomes and fibrosis were activated by transferring exosomes of AF patients (Supplemental Figure 5D and 5E). These results provided evidence that exosomes from AF patients could induce atrial inflammation and fibrosis to promote AF development.

## Discussion

In current study, our data reveals different circulating exosome lncRNAs between AF and SR individuals. Among these, six differential lncRNAs expressions in the plasma exosomes of SR controls and AF patients are verified. Our work also indicates that six circulating exosome lncRNAs could be effective biomarkers for distinguishing persistent AF and related to left atrial function. Besides, we provide evidence that these lncRNAs harbor a unique inflammatory function via regulating CXCR3 signaling pathway, which induces T cell infiltration into the atria and upregulated inflammatory, thereby leads to atrial structural remodeling. Collectively, these findings shed light on the role of circulating exosome lncRNAs as useful biomarkers of AF for diagnostic and prognostic evaluation.

Numerous studies have recognized that extracellular vesicles containing diverse biological contents such as lipids, proteins and various RNAs contribute to development and progression, and provide unique biomarkers in cardiovascular disease.^[18,19]^ Among these, exosomes are believed to transport into specific cells and then facilitate pathophysiological process, thereby constitutes an ideal therapeutic strategy.^[20]^ Although exosomes can be released into all types of biofluids, the blood circulation ones are the most widely explored for the easy accessibility and routine isolation.^[21]^ Previous data have revealed that the diagnostic role of circulating exosome proteins and miRNAs in diverse heart disease including coronary heart disease, hypertension and AF.^[22,23]^ Recently, lncRNAs have been identified as key molecules involved in the pathogenesis of AF by promoting vulnerate substrate, such as short refractory periods, slow conduction and atrial fibrosis.^[24]^ Several studies have stressed to identify circulating lncRNAs and their potential physiological roles in AF patients.^[25]^ However, the value of free circulating lncRNAs in AF diagnosis and prognosis is still limited. In the current study, circulating plasma exosomal lncRNAs between AF and SR patients were discovered and their potential effects to be potential biomarker and molecular mechanism in AF pathogenesis. In the present study, our data showed that 53 exosome lncRNAs differentially changed in AF condition, and six top lncRNAs were further verified in an amplified individual cohort. All of them were significantly increased in AF patients. Due to finite recording time window, a considerable number of silent or subclinical AF events would be missed in patients when only taking regular electrocardiogram or 24-hour Holter monitoring.^[26]^ Therefore, it is necessary to seek non-invasive methods for earlier diagnosing AF. Our study confirms the potential value of circulating exosome lncRNA in AF detection. Moreover, the expressions of NR046235, NR003045, NONHSAT167247.1 and NONHSAT200958.1 are increased in PeAF patients, and NONHSAT202361.1 and NONHSAT205820.1 perform lower levels. The AUCs of these 6 exosomal lncRNAs combination is higher than 0.8, which could efficiently distinguish paroxysmal from persistent AF. In sum, the combination of 6 circulating exosome lncRNAs can be used as potential biomarkers for molecular diagnosis, as well as enabling personalized medicine.

LA function and size have been recognized as reliable predictors of AF progression and adverse clinical outcomes.^[27]^ Left atrial enlargement can reflect increased filling pressure and hemodynamic abnormalities, which lead to thrombosis in left atrial appendage.^[28]^ At present, standard two-dimensional echocardiology and cardiac magnetic resonance imaging remain the most common detection for LA wall imaging and volumetric assessment in clinic.^[29]^ However, the image quality may be influenced at the onset of arrhythmia, and the cost of CMR is still high. Thus, specific biomarkers in diagnosis or prediction for atrial function and hemodynamics are considered promising in this field. The prevalence of NT-proBNP and C-protein reactive protein (CRP) were reported to define atrial diameter and function in patients with heart failure with preserved ejection fraction in AF.^[30]^ Though both markers could be effective prediction for LA enlargement, the levels would be affected by other diseases, such as chronic kidney dysfunction. Herein, our study describes an available role of circulating exosome lncRNAs, including NR046235, NONHSAT167247.1, NONHSAT202361.1 and NONHSAT205820.1, which are correlated with LA diameter and function evaluation. These findings would deepen our understanding about the pattern of circulating exosome lncRNAs in AF and provide potential biomarkers for clinical practice.

Increasing evidence undoubtedly support a functional interaction between lncRNA and atrial structure remodeling via inducing progressive cardiomyocyte death, fibroblast proliferation and inflammatory response.^[31]^ As a consequence, we established the network of target genes and then conducted further GO and KEGG analysis of these six lncRNAs, which reveals their probable biological processes in regulating genes responsible for AF pathogenesis. These exosome lncRNAs are mainly enriched in chemokine activity and CXCR chemokine receptor binding, especial for CXCR3 signaling pathway. As known, chronic subclinical inflammation defined as systemic immune response, is an important substrate for AF development. Similar to previous findings, our results demonstrate that CXCR3-related inflammatory response participate in cardiac remodeling especial for fibrosis.^[32]^ First, the plasma levels of CXCR3 related chemokines, including CXCL4, CXCL9, CXCL10 and CXCL11, are increased in AF patients. Moreover, highly CXCR3 expression, CD4^+^ and CD8^+^ T cells infiltration are found in atrial tissues of AF patients, which is positively correlated with fibrosis. CXCR3 is an interferon-inducible chemokine receptor that is widely expressed on monocytes, B cells, CD8^+^T cells, NK cells and dendritic cells. CXCR3 recruits T cells to the peripheral inflammatory sites, which leads to an optimal humoral response. In pathogenesis of diverse CVDs, such as hypertension and heart failure, CXCR3 promotes T cell polarization into effector Th1/Th17 cells, macrophage activation and Treg infiltration, which exacerbates pro-inflammatory response and cardiomyocytes injury.^[33]^ Our findings uncover that exosome lncRNAs may drive CXCR3-mediated inflammatory response and lead to atrial structural remodeling and AF development. Future studies are warranted to address this possibility and the potential molecular mechanism.

## Conclusion

In summary, our current study firstly characterizes the features of circulating exosome lncRNAs in AF patients, and identifies 6 significantly differentiated plasma exosomes lncRNAs as effective biomarkers for diagnosis of persistent AF. These exosomes lncRNAs regulate CXCR3 signaling pathway to drive pro-inflammatory profiles and exacerbate atrial remodeling. These findings provide a new perspective that circulating exosome lncRNAs play critical roles in the process of AF and facilitate the knowledge of exosomes-based diagnosis and therapeutic targets.

## Supplementary Material

Supplementary Material
