# Supplementary material for: Circulating exosome long non-coding RNAs are associated with atrial structural remodeling by increasing systemic inflammation in atrial fibrillation patients
Source: J Transl Int Med. 2024 Mar 21;12(1):106–18. doi: 10.2478/jtim-2023-0129 (PMC10956728; doi:10.2478/jtim-2023-0129)
Supplement: Supplementary file 1 — Supplementary Material [file jtim-2023-0129_sm.pdf]

METHODS AND MATERIALS

Patients in the study

The diagnosis of AF *via* the medical history, 12-lead electrocardiogram and 24 h electrocardiogram monitoring. The AF group contained paroxysmal and persistent AF patients, without permanent AF. We identified that paroxysmal AF returns to regular heartbeat within 7 days on its own or with treatment, and persistent AF last longer than seven days and cannot be able to regulate itself anymore. Patients attending cardiology department for non-AF-related conditions were screened and selected to create a sinus rhythm (SR) group. Some patients were excluded from SR group if they had any previous AF or other arrhythmia history. The patients of two groups were excluded if they had severe chronic kidney disease (eGFR < 30 mL/min), recent or active tumor, thyroid disease, acute myocardial infarction, stroke, other types of arrhythmias and acute heart failure. Finally, we selected 105 SR individuals and 110 AF patients enrolled in the current study. We also collected peripheral blood samples to obtain plasma *via* centrifuge (3500 rpm/min, 15 min, 4°C), then stored in-80°C freezer for further experiments. Besides, we harvested 15 atrial samples from patients accepting mitral valve replacement surgery, including 8 persistent AF patients and 7 individuals with SR. The atrial tissues were frozen immediately by liquid nitrogen during the surgery and then stored at-80°C.

Isolation, purification and identification of Exosomes

The methods of isolation, separation and purification in circulating exosomes were guided by recent protocols.[1] Briefly, plasma samples were centrifuged at 300× g for 10 min, 2, 000× g for 30 min and 10, 000× g for 60 min. Then the supernatant was followed by filtration through a 0.22 μm filter to eliminate cellular debris. For further purification, the supernatant was ultracentrifuged at 120, 000× g for 90 min twice. The procedures of centrifugation were performed at 4°C. The exosome pellet was resuspended in 50 μL PBS. Then the morphology of exosome was observed by transmission electron microscope (EM, HT-7700, Hitachi, Japan). The expressions of exosomal specific surface markers including CD81 and Alix were measured *via* Western blot.

Exosome labeling

Exosomes were labeled with PKH26 Fluorescent Cell Linker Kit (Sigma-Aldrich, USA). Resuspended plasma exosomes were added to 150 μL of Diluent C (Sigma-Aldrich, USA). Then 2 μL of PKH26 dye was added to 500 μL of Diluent C and incubated with the exosome-Diluent C solution for 5 min at room temperature. The labeled exosomes were purified *via* centrifugation to remove the excess dye and resuspended in 100 μL of PBS for further experiments. Cardiomyocytes were stained by WGA. The uptake of labeled exosomes by cardiomyocytes was visualized by confocal microscopy (Zeiss, Germany).

Western blot

The detailed method of Western blot was referred to our previous study. [2] Whole protein of exosomes was extracted and resolved on 10% SDS-PAGE and then transferred on 0.45 μm polyvinylidene fluoride membrane. The membrane was blocked with 5% BSA for 1 h at room temperature, then incubated with primary antibody at 4°C overnight. The membrane was incubated with related secondary antibody for 1 h. The expressions of proteins were analysis by ChemiDoc XRS gel documentation system *via* ECL kit (Thermo). Primary antibody applicated in western blot as follow: CD81, Proteintech 99866-1-Ig, Mouse, 1: 1000; Alix, Proteintech 12422-1-AP, Rabbit, 1: 1000; GAPDH, CST #5174, Rabbit, 1: 1000, NLRP3, Proteintech 68102-1-lg, Caspase 1, Proteintech 22915-1-AP, CXCR3, Proteintech 26756-1-AP, Collagen 1, Proteintech 14695-1-AP, α-SMA, Proteintech 67735-1-lg.

Real-Time Quantitative Reverse Transcription (qRT-PCR)

Total RNAs of circulating exosomal lncRNAs and rat atrial tissues were isolated by kits (Axygen, USA). Then the RNAs were transferred into cDNAs by kits (Tyobo 301). qRT-PCR was performed on Applied Bio-system. The relative levels of lncRNAs and mRNAs were calculated and quantified using $2^{-\Delta\Delta CT}$ method after normalization with GAPDH. The sequences of lncRNAs and GAPDH primers: NR046235 (Forward, GTCTCCTCTGACTTCAACAGCG; Reverse, ACCACCCTGTTGCTGTAGCCAA), NR003045 (Forward, GTGTGTGGGTTGACTTCGGA; Reverse, AAGGCTTTTCTCACCGAGGG), NONHSAT167247.1 (Forward, TAGCTGCTGTCCCCAAACAC; Reverse, CCTCAAAGTGCCATACCGCT), NONHSAT202361.1 (Forward, GGAGTCCTATGCAGCAACCA; Reverse, AAGTGGGCTCCCTCTAGTGT), NONHSAT205820.1 (Forward, GAAGTCAAGGGCAGACCCTC; Reverse, CAGTCAAGCCAGGGACTGTT), NONHSAT200958.1 (Forward, TTACCGACGTCCCTCAAGAC; Reverse, ACTCCTGGGAAACTCTTGGGA), GAPDH (Forward, GTCTCCTCTGACTTCAACAGCG; Reverse, ACCACCCTGTTGCTGTAGCCAA).

Pathological staining

Human atrial tissues were fixed in 4% paraformaldehyde. HE, Masson's trichrome and immunohistochemistry staining were performed as previous study[2]. All information of primary antibodies was as follows: CD3, Proteintech

(17617-1-AP), 1: 200; CD4, CST (#48274), 1: 100; CD8, Proteintech (66868-1-Ig), 1: 200; CD68, Proteintech (66231-2-Ig), 1: 200.

Inflammation markers

Plasma levels of four chemokines including CXCL4, CXCL9, CXCL10 and CXCL11 were detected by Elisa kits according to the manufacturer's instructions. The information was as follows: CXCL-4, Human, CSE-E07882h, range 20-15000 μg/L; CXCL-9, Human, ab100595, range 8.23-6000 ng/L; CXCL-10, Human, ab83700, range 6.25-200 ng/L; CXCL-11, Human, ab289695, range 31.25-2000 ng/L.

Transthoracic Echocardiograph and Cardiac Magnetic Resonance

All patients had transthoracic echocardiography with tissue Doppler analysis performed by board certified physicians. LA diameter, RA diameter and LVEF were recorded. Cardiac magnetic resonance (CMR) detection was performed on a 3.0 T scanner according to standard protocols.[3] Left atrial appendage flow velocity (LAAFV) of AF individuals were measured by CMR.

REFERENCES

1. Bordanaba-Florit G, Royo F, Kruglik SG, Falcón-Pérez JM. Using single-vesicle technologies to unravel the heterogeneity of extracellular vesicles. Nat Protoc 2021;16:3163–3185.
2. Yuan Y, Zhao J, Gong Y, Wang D, Wang X, Yun F, *et al*. Autophagy exacerbates electrical remodeling in atrial fibrillation by ubiquitin-dependent degradation of L-type calcium channel. Cell Death Dis 2018;9:873.
3. Kramer CM, Barkhausen J, Flamm SD, Kim RJ, Nagel E; Society for Cardiovascular Magnetic Resonance Board of Trustees Task Force on Standardized Protocols. Standardized cardiovascular magnetic resonance (CMR) protocols 2013 update. J Cardiovasc Magn Reson 2013;15:91.

Supplemental Figure 1

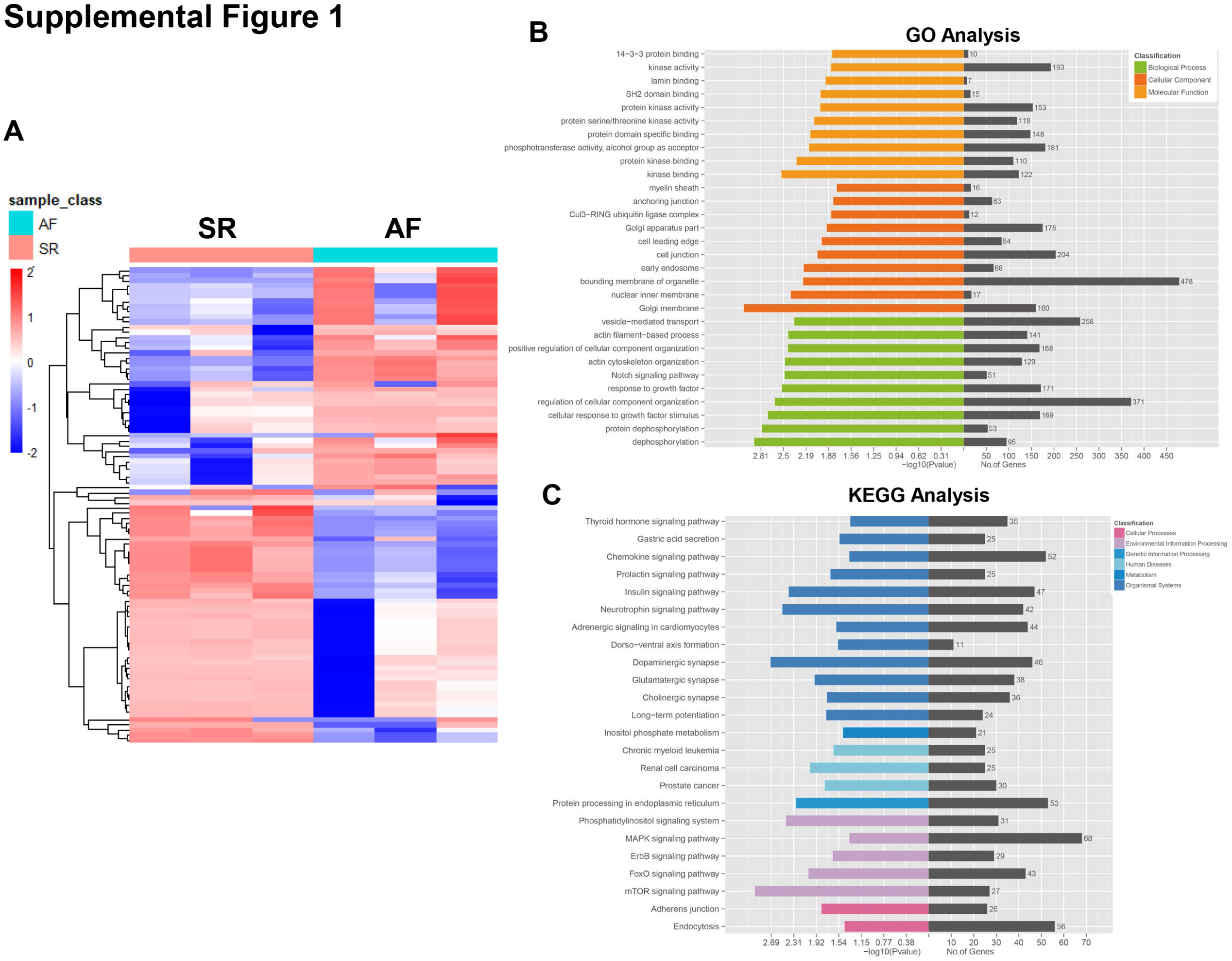

Supplemental Figure 1: Profile of differential circulating exosome mRNAs in AF. (A) Heatmap of differential circulating exosome mRNAs between SR controls (n = 3) and AF patients (n = 3). (B) GO functional analysis of differential circulating exosomal mRNAs. (C) KEGG pathway analysis of differential circulating exosomal mRNAs. n represents the number of patients in every group. SR: sinus rhythm, AF: atrial fibrillation.

Supplemental Figure 2

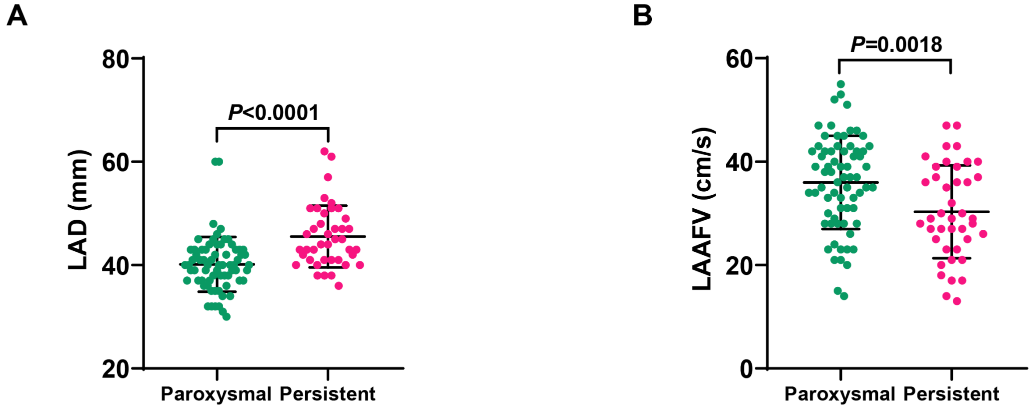

Supplemental Figure 2: LAD and LAAFV of AF patients. (A) LAD of paroxysmal (n = 69) and persistent AF patients (n = 41) tested by Echocardiography. (B) LAAFV of paroxysmal (n = 69) and persistent AF patients (n = 41) tested by CMR. LAD: left atrial diameter, LAAFV: left atrial appendage flow velocity, CMR: cardiac magnetic resonance. *n* represents the number of patients in every group.

Supplemental Figure 3

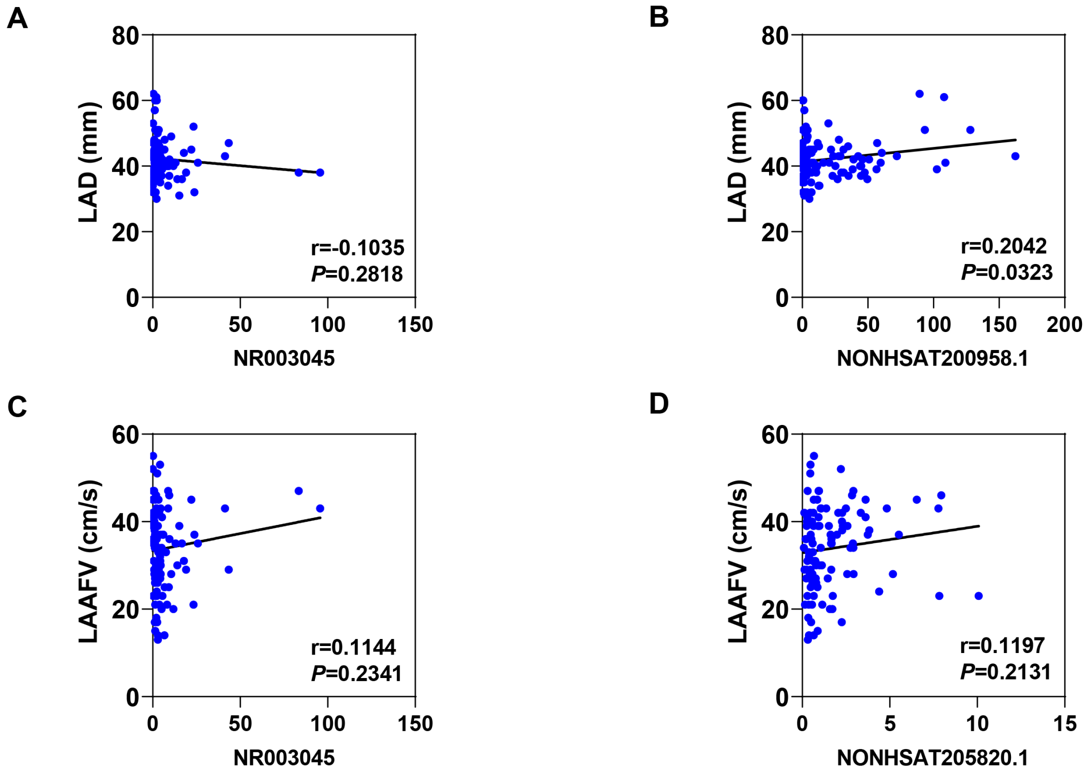

Supplemental Figure 3: Negative correlation between several exosomal lncRNAs and atrial structure in AF patients. (A) Pearson analysis of circulating exosomal NR003045 correlated with LAD in AF patients, r = -0.1035 and P = 0.2818. (B) Pearson analysis of circulating exosomal NONHSAT200958.1 correlated with LAD in AF patients, r = 0.2042 and P = 0.0323. (C) Pearson analysis of circulating exosomal NR003045 correlated with LAAFV in AF patients, r = 0.1144 and P = 0.2341. (D) Pearson analysis of circulating exosomal NONHSAT205820.1 correlated with LAAFV in AF patients, r = 0.1197 and P = 0.2131. LAD: left atrial diameter, LAAFV: left atrial appendage flow velocity.

Supplemental Figure 4

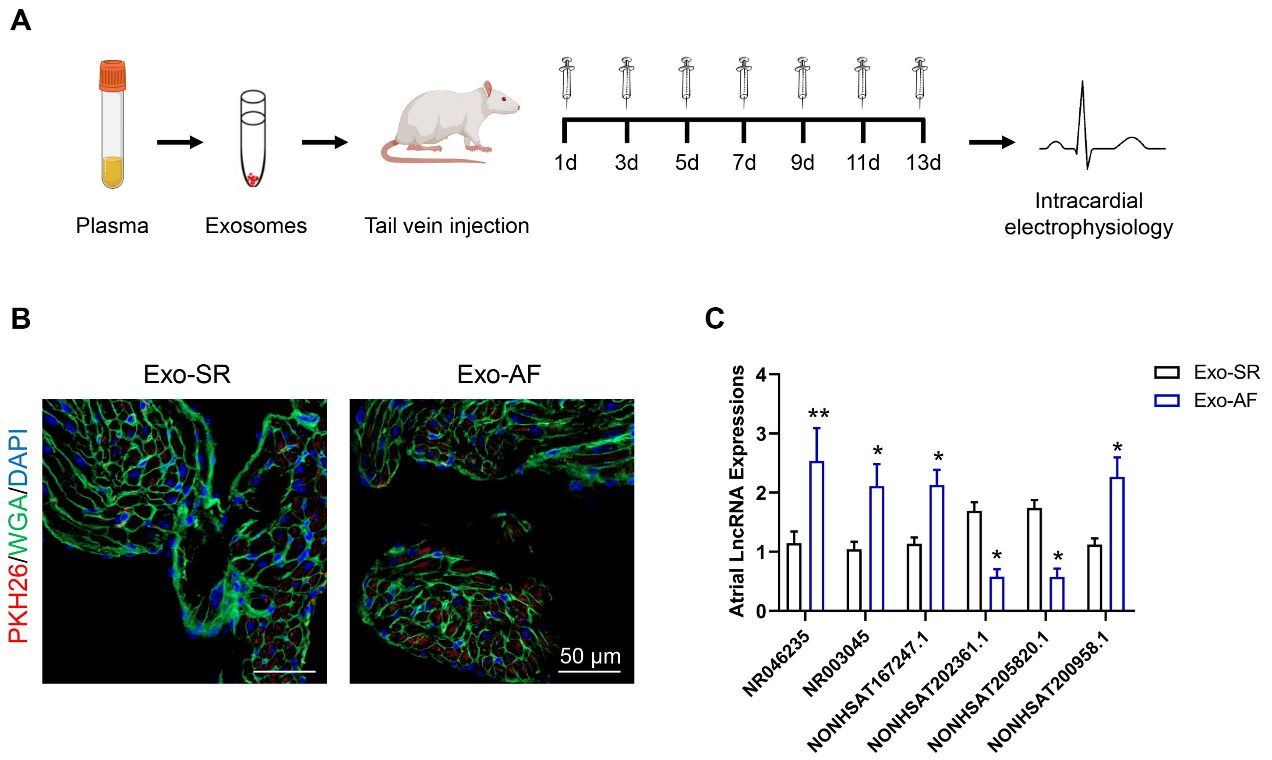

Supplemental Figure 4: Circulating exosomes from human plasma can be absorbed by atrial cardiomyocytes *in vivo*. (A) Scheme for human plasma exosomes preparation, transplant and AF induction in rats. (B) PKH26 and WGA staining in Exo-SR and Exo-AF rats, scale bar 50 μm (*n* = 3/group). (C) The lncRNA expressions in the atria of rats (*n* = 6/group).

Supplemental Figure 5

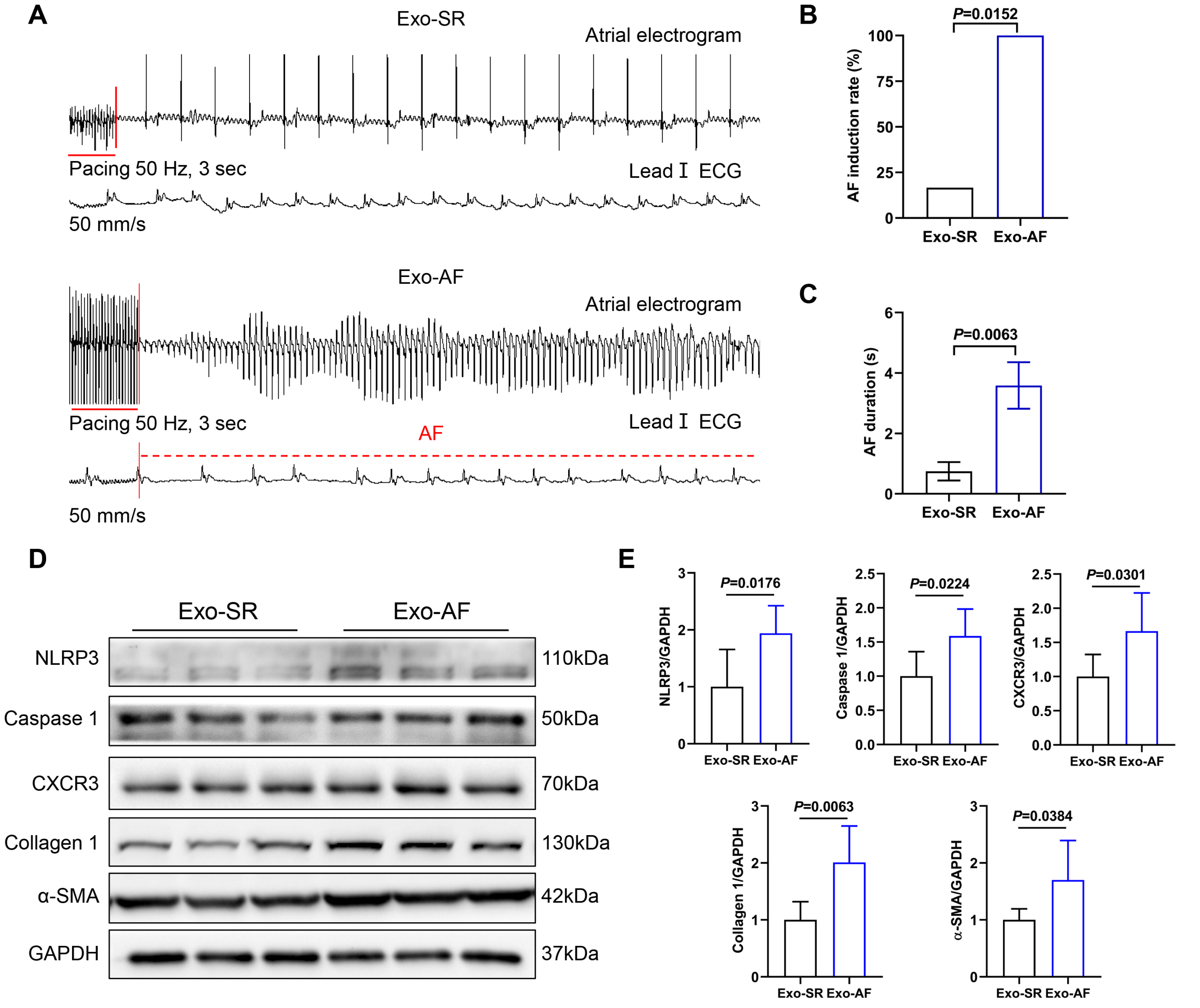

Supplemental Figure 5: Circulating exosomes from human plasma enhances AF susceptibility in rats. (A) Representative trace of simultaneous recordings of surface ECG (lead I) and intracardiac electrograms in Exo-SR and Exo-AF rats after programmed intracardiac stimulation (red line). (B) Incidence of pacing-induced AF in Exo-SR and Exo-AF rats (n = 6/group). (C) AF duration in Exo-SR and Exo-AF rats (n = 6/group). (D and E) NLRP3, Caspase 1 and CXCR3 protein levels in the atrium of rats (n = 6/group), Collagen 1 and α–SMA protein levels in the atrium of rats (n = 6/group).

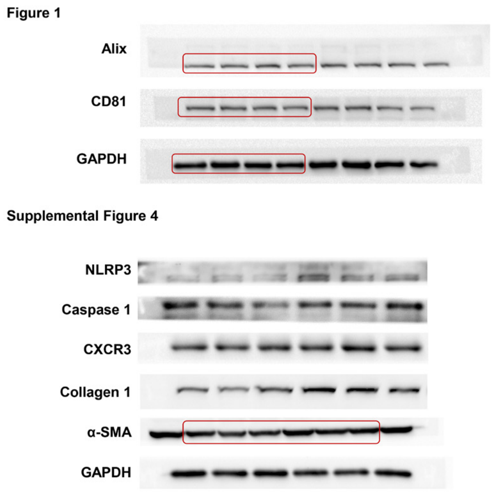

Supplemental Figure 6. Original bands.

Supplemental Table Clinical characteristics of 6 individuals with plasma exosomal lncRNA sequencing

Characteristics	SR group (*n* = 3)	AF group (*n* = 3)	*P* value
Age	55.67 ± 1.16	62.00 ± 5.29	0.1128
Gender, *n*			
Male	2	2	
Female	1	1	
BMI (kg/m^2)	26.46 ± 4.25	24.44 ± 2.66	0.5229
Smoking, *n*	3	2	
Hypertension, *n*	2	3	
Diabetes, *n*	2	1	
Coronary heart disease, *n*	3	2	
Heart Failure, *n*	0	1	
Chronic kidney disease, *n*	0	0	
Aspirin	3	2	
Statin	2	1	
β-receptor blocker	1	1	
ACEI/ARBs	0	2	
CCB	0	0	
NOAC	0	1	
LAD (mm)	35.33 ± 1.16	46.00 ± 6.93	0.0582
RAD (mm)	39.33 ± 1.16	39.33 ± 3.06	0.9999
LVEF (%)	66.33 ± 5.51	55.33 ± 6.81	0.0952

BMI, body mass index; ACEI, angiotensin enzyme inhibitor; ARBs, angiotensin receptor blockers; CCB, calcium channel blocker; NOAC, novel oral anticoagulant. TG, triglyceride; CHOL, cholesterol; Cr, creatinine; UA, uric acid; NT-proBNP, N-terminal pro-B-type natriuretic peptide; LAD, left atrial diameter; RAD, right atrial diameter; LVEF, left ventricle ejection fraction.

Supplemental Table Differential exosomal lncRNAs

transcript	Control_1	Control_2	Control_3	Disease_1	Disease_2	Disease_3	log2(Fold_change)	p-value	q-value
NONHSAT167247.1	0	0	1.47862	30.08255	35.0892	25.0759	5.34660362699995	9.40378295766376E-09	0.0000419661898683741
NR_003045	16.2808	6.53935	11.41008	25.9792	317.506	171.7426	3.9118677581136	1.98431180944898E-35	2.10208071533977E-31
NONHSAT200958.1	2.617395	4.0971	1.13769	26.4049	33.5156	19.2942	3.33460210972545	1.76117270549653E-06	0.000632659037209789
NONHSAT195005.1	1.89697	0	3.79394	17.6117	16.2994	18.924	3.21476540627895	0.000121195471489591	0.00656810394999402
NR_046235	2.44628	3.82925	1.06331	19.3088	17.4025	21.2151	2.98059705811938	0.000100081284271509	0.00588567228282979
NONHSAT248600.1	2.997165	1.10131	4.89302	23.48125	14.0141	32.9484	2.96983879643438	0.0000184452325891582	0.00233391530787353
NONHSAT215901.1	2.79313	1.73408	0	19.21455	13.85218	38.4291	2.78224456192305	0.000174418553919543	0.00821338669289147
NONHSAT223708.1	2.41125	3.77441	1.04809	16.5654	20.584	12.5468	2.78031989679913	0.000494167524781528	0.015306529071116
NR_002988	3.958005	11.45883	5.095531	23.86365	59.78552	69.5433	2.591969383	0.00000469	0.000904566
NONHSAT217140.1	3.090805	2.92866	3.25295	17.15056	31.056	23.24512	2.47220114200802	0.000862869771837797	0.0211981324637602
NONHSAT175406.1	6.945555	10.1377	3.75341	37.15065	36.8576	37.4437	2.41922556717469	1.26707142980006E-06	0.000542502944648345
NONHSAT185643.1	2.862845	2.71266	3.01303	14.57365	17.8756	11.2717	2.34784078128952	0.00285165863318214	0.0429432772495942
NONHSAT121068.2	5.60556	9.17331	2.03781	23.89843	9.17165	38.6252	2.09198513032355	0.00036133489006859	0.012697058538661
NONHSAT247447.1	4.1776	1.92863	6.42657	17.72187	33.3067	12.13703	2.08478416923751	0.00217679567646852	0.0366048699044409
NONHSAT233298.1	4.167435	1.92394	6.41093	17.52889	29.7282	10.32958	2.07250303911571	0.00238827470604912	0.03876227642228
NONHSAT235143.1	682.059	1677.06	3416.42	5155.78	13195.7	6938.8795	2.02221736859903	6.91817537402229E-219	1.4657538164941E-214
NONHSAT167166.1	4.27097	8.54194	0	17.1074	16.6371	17.5777	2.0019848448326	0.00332213081664499	0.0470310930521375
ENST00000434611.1	9.890395	4.43012	19.39444	38.06408	54.9851	69.04235	1.944330164	0.0000000000000273	0.0000000000328
NONHSAT163033.1	6.471495	7.43642	5.50657	24.29595	10.1387	38.4532	1.90854490735874	0.000688479276378945	0.0185045750378154
ENST00000365494.1	295.823	588.36	442.0915	1358.1	1935.74	1646.92	1.89735357582235	9.61806535588176E-146	1.528334630213E-141
ENST00000584923.1	139.4663	218.4478	129.8852	516.7935	682.3433	724.42511	1.889671367	0.000000000000000222	0.000000000000478
ENST00000580625.1	11091.9	9744.83	10418.37	13154	57346.4	35250.2	1.75850255611176	0	0
ENST00000553637.1	10981	9650.83	10315.92	13022.4	56775	34898.7	1.75830150515652	0	0
NONHSAT238587.1	6.84625	0	0	21.7795	43.559	13.6925	1.66958495358734	0.00328093641018687	0.0466642622792329
NONHSAT164843.1	12.64625	10.9646	14.3279	39.81425	41.0364	38.5921	1.65457523602111	0.0000779175605732778	0.0050563616070008
NONHSAT183545.1	7.97879	6.84031	9.11727	24.28935	27.3563	21.2224	1.60608200249908	0.00250181795531395	0.0397597503568111
NONHSAT223878.1	8.6821	17.3642	0	24.96977	18.49843	41.4411	1.52406629888617	0.00312883086483592	0.0454195227382599
ENST00000588763.1	42.6648	27.9286	57.401	96.34315	94.4735	98.2128	1.175135809	0.0000261	0.039209679
ENST00000363046.1	884.701	813.806	955.596	1159.135	1261.47	1056.8	0.389786742	0.00000807	0.022293131
NR_003051	881.753	813.177	950.329	1153.945	1258.47	1049.42	0.388127979	0.00000948	0.023619554

(To be continued)

(Continued)

NONHSAT201387.1	36.34995	20.9337	55.4265	10.46685	0	17.2734	-1.79612605138315	0.000152666026251825	0.00753139414370715
ENST00000461943.1	15.6973	44.9053	52.9541	4.06781	5.0983	3.86758	-1.948192231	0.00000241	0.00055617
NONHSAT187578.1	24.39575	34.3285	14.463	5.69998	4.8417	6.55826	-2.0976010745123	0.000557060269689518	0.0164300211215238
NONHSAT168349.1	20.20719	17.88487	32.5295	4.607205	9.21441	0	-2.13290466040817	0.0014930348309511	0.0293771123342812
NONHSAT150378.1	41.7892	13.9337	69.6447	9.302745	3.16619	15.4393	-2.1674017541689	3.92668799735813E-06	0.00100355420337767
NONHSAT247960.1	26.10095	20.7593	31.4426	5.61574	6.00366	5.22782	-2.21655426995608	0.000215315596040212	0.00929429635734591
NONHSAT186557.1	17.86827	26.8047	8.93184	3.65453	7.30906	0	-2.28964217807871	0.00177839424076082	0.0325468586365107
NONHSAT154179.1	18.21397	14.99414	31.4338	3.57023	5.29586	1.8446	-2.35095648987073	0.00134113965040844	0.0275420236525472
NONHSAT222552.1	18.3545	22.7879	13.9211	3.56065	2.07125	5.05005	-2.36592127843894	0.00122972516218905	0.0261759329607036
NONHSAT172378.1	19.15097	13.36963	34.9323	3.51007	2.04183	4.97831	-2.44784538304322	0.000752451308628577	0.0196195337843087
NR_036211	25.1255	28.033	26.57925	1.59097	7.26734	4.429155	-2.58519700504764	0.0000140488690554128	0.0330725987418924
NONHSAT100866.2	15.36775	23.0536	7.6819	2.095405	4.19081	0	-2.87460493461462	0.000960976020677694	0.0226124615046102
NONHSAT246710.1	11.83305	7.3464	16.3197	1.48004	0	2.96008	-2.9991139078824	0.00307408875704463	0.0449001156193214
NONHSAT164539.1	22.69655	27.7684	17.6247	2.535415	3.60564	1.46519	-3.16217920277211	0.0000260311067562445	0.00280966448086237
NONHSAT128417.2	14.99828	11.94285	28.0537	1.517867	0.882953	2.15278	-3.30467976829497	0.000486653110209792	0.0151668977646098
NONHSAT139444.2	10.70885	5.76899	19.6487	0	0	1.96014	-3.44977428152015	0.00268085488158065	0.0414690830435678
NONHSAT202591.1	20.5048	28.7849	12.2247	1.791901	0.769512	2.81429	-3.51639883410898	0.0000282758395003646	0.00291630725086872
NONHSAT205820.1	28.69285	10.5432	46.8425	1.947075	0	3.89415	-3.8813109131963	2.77148406414935E-07	0.000236452423502389
NONHSAT202361.1	29.83475	13.3458	46.3237	1.848485	0	3.69697	-4.01257844632352	1.17650796701071E-07	0.000143694967802371
NONHSAT249508.1	12.37429	18.322	6.42657	0	0	1.06852	-4.5336593500943	0.000421173724797348	0.0139108418127629
NONHSAT257333.1	19.22375	16.0285	22.419	0	0.539583	0.657795	-5.00493921843688	7.59934135835998E-06	0.00141080252449681
NONHSAT224496.1	17.0928	30.7681	3.4175	0	0	0	-5.09531684153477	0.0000232706145975741	0.00264455378232764
NR_046944	20.579	17.2203	18.89965	0	0	0	-5.2402876125115	4.34610881331134E-06	0.0131544296325182
